# Extended DaraKRd: Are we enhancing outcomes by prolonging treatment?

**DOI:** 10.1038/s41408-024-01070-2

**Published:** 2024-06-04

**Authors:** Timothy M. Schmidt

**Affiliations:** University of Wisconsin Carbone Cancer Center, Madison, WI USA

**Keywords:** Myeloma, Cancer immunotherapy, Combination drug therapy, Phase II trials

In the manuscript that accompanies this editorial, Derman, et al. report the final analysis of a phase 2 clinical trial of extended daratumumab, carfilzomib, lenalidomide, and dexamethasone (DaraKRd) without transplant for patients with newly diagnosed multiple myeloma (MM) [1]. Patients were treated with the quadruplet regimen for two years, followed by lenalidomide maintenance per standard of care. The study easily met its primary endpoint, with 75% of patients achieving a stringent complete response and/or measurable residual disease (MRD) negativity at <10^−5^ after cycle 8 of therapy, and more than half of patients had undetectable MRD ( < 10^−6^) at any time point. With a median follow up of 27 months, the 3-year progression-free survival (PFS) and overall survival were estimated at 85% and 95%, respectively. The regimen was described as well-tolerated, given that no patients discontinued study treatment due to toxicity.

These outstanding results add to the growing body of literature demonstrating the profound efficacy of CD38 antibodies, proteasome inhibitors (PI), immunomodulators (IMiD), and steroids for treating newly diagnosed MM, with a majority of patients with newly diagnosed MM achieving durable disease control regardless of how these three drug classes are utilized. However, such rapid progress can sometimes feel like a double-edged sword, as it is challenging to incorporate the plethora of new data into the management of a complex, heterogeneous disease for which a reliable cure remains stubbornly elusive.

When considering quadruplet therapy for patients with newly diagnosed myeloma, the primary considerations are (a) patient fitness (b), which PI to use (if any) (c), whether to proceed with autologous stem cell transplant (ASCT), and (d) do these choices affect the type and/or duration of maintenance therapy. How should we incorporate the results for extended DaraKRd into the treatment algorithm for patients with newly diagnosed MM?

First, we must recognize that, although the trial did not utilize ASCT, the population comprised a group of patients that would generally be considered as “transplant eligible”. The median age was 58, with <30% of patients enrolled being over the age of 65 and 88% of patients had stem cells collected. As such, extreme caution must be taken if applying these data to unfit or frail patients. In fact, despite excellent response rates, elderly patients have often experienced less long-term benefit from PI-based regimens, as noted in S0777 [2], PERSEUS [3], and particularly the GEM2017FIT trial, which showed higher mortality rates for patients aged 65-80 who were treated with DaraKRd [4]. In contrast, the addition of daratumumab to Rd was shown to have a profound PFS benefit among unfit and frail patients in the MAIA trial, without any early mortality concerns [5]. As such, DaraRd seems to be an effective and well tolerated combination for nearly all patients, and whether to incorporate a PI with initial therapy at all is a question which is being investigated by the ongoing Phase 3 ECOG EAA181 (EQUATE) trial, with additional context to be provided by the ongoing CEPHEUS (NCT03652064) and IMROZ (NCT03319667) trials.

Due to the single-arm, phase 2 design of the present study we cannot determine whether extended DaraKRd is preferred over other treatment strategies for transplant-eligible MM, which might include a more abbreviated course of the same regimen followed by less intensive maintenance therapy, an alternative choice of CD38 antibody, PI, or IMiD, and/or the use of consolidative autologous transplant.

The clearest insight gained from this study is context to assist with shared decision making in the clinic, regarding expectations of continuing quadruplet therapy without ASCT. It is clearly feasible to administer DaraKRd for two years, with deepening of some patients’ responses over time. Stem cell mobilization and collection were shown to be feasible with the use of upfront plerixafor and G-CSF, in contrast to what was observed in the IFM 2018-04 trial [6]. MRD-negativity rates and PFS are similar to what has been reported in other trials using a CD38 antibody plus KRd with or without ASCT, a list of which are detailed in Table 1. As such, this type of regimen could be considered an alternative to the standard recommendation for quadruplet induction followed by consolidative ASCT. However, given that long-term outcomes seen in this trial are similar to others with a shorter period of intensive therapy, especially with a twice-weekly carfilzomib schedule that many patients would find undesirable and practically difficult, it is difficult to recommend this particular strategy for most patients, in the absence of head-to-head data.

**Table 1:** Published trials using a CD38 antibody, carfilzomib, lenalidomide, and dexamethasone for newly diagnosed multiple myeloma.

Trial	Dose/schedule	N	Age	High risk	Med f/u	MRD- rates	PFS	OS
Derman et al. [1]	DaraKRd × 24 cycles (96w) -> R mt Dara: 16 mg/kg IV d1/8/15/22 (c1-2), d1/15 (c3-6), d1 (c7 + ) K: 20/36 mg/m2 IV d1/2/8/9/15/16 (c1-8), 1/2/15/16 (c9-24) R: 25 mg PO d1-21 d: 40 mg PO/IV d1/8/15/22	42	58 (39–79)	Any: 24 (57%) 2 + HRCA: 10 (24%)	27 m	After 8c: 10^-5^: 59% 10^-6^: 35% Any time: 10^-5^: 65% 10^-6^: 53%	36 m PFS Overall: 85% 2 + HRCA: 60%	36 m OS Overall: 95%
MANHATTAN [10]	DaraKRd x 8 cycles (32w) -> SoC Dara: 16 mg/kg IV d1/8/15/22 (c1-2), d1/15 (c3-6), d1 (c7 + ) K: 20/56 mg/m2 IV d1/8/15 R: 25 mg PO d1-21 d: 40 mg PO/IV d1/8/15/22	41	59 (30–70)	20 (49%)	20.3 m	After 8c: 10^-5^: 71% 10^-6^: NR	12 m PFS Overall: 98%	12 m OS Overall: 100%
MASTER [11]	DaraKRd x 4c (16w) -> ASCT -> [DaraKRd x 4-8c per MRD] Dara: 16 mg/kg IV d1/8/15/22 (c1-2), d1/15 (c3-6), d1 (c7 + ) K: 20/56 mg IV d1/8/15 R: 25 mg d1-21 d: 40 mg 1/8/15/22	123	61 (35–79)	Any: 70 (59%) 2 + HRCA: 24 (20%)	42.2 m	Any time: 10^-5^: 72% 10^-6^: 71%	36 m PFS 0 HRCA: 88% 1 HRCA: 79% 2 HRCA: 50%	36 m OS 0 HRCA: 94% 1 HRCA: 92% 2 HRCA: 75%
IFM 2018-04 [6]	DaraKRd x 6c -> ASCT1 -> DaraKRd x 4c -> ASCT2 - > DR mt Dara: 16 mg/kg IV d1/8/15/22 (c1-2), d1/15 (c3-10), q8w (mt) K: 20/36 mg/m2 IV d1/2/8/9/15/16 (c1-6), 1/8/15 (c7-10) R: 25 mg d1-21 (c1-6); 15 mg d1-21 (c7-10), 10 mg d1-21 (mt) d: 20 mg d1/2/8/9/15/16/22/23 (c1-6), 40 mg d1/8/15/22 (c7-10)	50	57 (38–65)	Any: 50 (100%) 2 + HRCA: 30 (60%)	32 m	After ASCT2: 10^-6^: 94%	30 m PFS Overall: 80%	30 m OS Overall: 91%
GEM-2017FIT [4]	DaraKRd x 9 cycles (36w) -> DK x 9 cycles (36w) Dara: 1800 mg SC d1/8/15/22 (c1-2), d1/15 (c3-6), d1 (c7-18) K: 36 mg/m2 IV d1/2/8/9/15/16 (c1-2), 56 mg/m2 d1/8/15 (c3-18) R: 25 mg PO d1-21 every 28 days (c1-9) d: 40 mg PO d1/8/15/22 (c1-9)	153	73 (66–80)	Any: 53 (54%)	33 m	After 18c 10^-5^: 84% 10^-6^: 79%	30 m PFS 79%	18 m OS Overall: 90%
GMMG-CONCEPT (TE) [12]	IsaKRd x 6c -> ASCT -> IsaKR x4c -> IsaKR x26c Isa: 10 mg/kg IV d1/8/15/22 (c1) d1/15 (c2 + ) K^a^: 20/56 mg/m2 IV d1/8/15 (c1-10), 70 mg/m2 d1/15 (c11 + ) R: 25 mg PO d1-21 (c1-10), 15 mg d1-21 (c11 + ) d: 40 mg d1/8/15/22 (c1-10)	99	58 (35–73)	Any: 99 (100%) 2 + HRCA: 31 (31%)	44 m	Any time 10^-5^: 82% Post-cons 10^-5^: 68%	36 m PFS Overall: 69%	24 m OS Overall: 84%
GMMG-CONCEPT (TIE) [12]	IsaKRd x 8c -> IsaKR x4c -> IsaKR x26c Isa: 10 mg/kg IV d1/8/15/22 (c1) d1/15 (c2 + ) K^a^: 20/56 mg/m2 IV d1/8/15 (c1-12), d1/15 (c13 + ) R: 25 mg PO d1-21 (c1-12), 15 mg d1-21 (c13 + ) d: 40 mg d1/8/15/22 (c1-12)	26	74 (64–87)	Any: 26 (100%) 2 + HRCA: 7 (27%)	33 m	Any time 10^-5^: 69% Post-cons 10^-5^: 54%	36 m PFS Overall: 58%	24 m OS Overall: 71%
IsKia [13]	IsaKRd x 4c -> ASCT -> IsaKRd x4c -> IsaKRd x12c Isa: 10 mg/kg IV d1/8/15/22 (c1), d1/15 (c2-8), d1 (c9 + ) K: 20/56 mg/m2 IV d1/8/15 (c1-8), d1/15 (c9 + ) R: 25 mg PO d1-21 (c1-8), 10 mg d1-21 (c9 + ) d: 40 mg d1/8/15/22 (c1-8), 20 mg d1/15 (c9 + )	151	61 (55–66)	Any: 62 (41%) 2 + HRCA: 13 (9%)	20 m	After ASCT 10^-5^: 64% 10^-6^: 52% After c8 IsaKRd 10^-5^: 77% 10^-6^: 67%	12 m PFS Overall: 95%	Not reported
SKylaRk [14]	IsaKRd x4c -> ASCT + IsaKRd x2c OR IsaKRdx4 -> IsaR +/- K mt Isa: 10 mg/kg IV d1/8/15/22 (c1-2), d1/15 (c3-6), d1 (c7 + ) K: 20/56 mg/m2 IV d1/8/15 R: 25 mg PO d1-21 d: 20 mg d1/2/8/9/15/16/22/23 (c1-2), 20 mg d1/2/8/9/15/16 (c3 + )	50	59 (40–70)	Any: 23 (46%) 2 + HRCA: 5 (10%)	26 m	After c4 10^-5^: 43% 10^-6^: 18% Post-cons 10^-5^: 66% 10^-6^: 17%	24 m PFS Overall: 91.3%	24 m OS Overall: 95.8%

*Dara* daratumumab, *Isa* isatuximab, *K* carfilzomib, *R* lenalidomide, *d* dexamethasone, *c* cycles, *cons* consolidation, *mt* maintenance, *HRCA* high risk cytogenetic abnormalities. ^a^After a protocol amendment. Initial carfilzomib dosing was 20/36 mg/m^2^ twice-weekly.

At first glance, extended DaraKRd might seem appealing for patients who are at a high risk of relapse, namely those with ultra-high risk disease, defined by the presence of two or more high risk cytogenetic abnormalities, extramedullary disease, and/or plasma cell leukemia. However, despite continuous use of aggressive multi-agent therapy, too many patients with these features continue to relapse early, a pattern which has been demonstrated now in multiple trials using quadruplets and ASCT [7]. Derman et al. astutely highlight in their discussion that these results support the conclusion that patients with ultra-high risk disease must be identified early so that they may be considered for enrollment in clinical trials with investigational approaches beyond repurposing and/or extending the use of these 3 drug classes.

One of the most intriguing aspects of this manuscript is its comprehensive implementation of peripheral blood (PB) and bone marrow (BM) based MRD assessments throughout the duration of study therapy. Two different PB assays to detect low-level monoclonal protein by matrix-assisted laser desorption/ionization time-of-flight (MALDI-TOF) and the more sensitive liquid chromatography-mass spectrometry (LC-MS), were explored for prognostic purposes as well as concordance with marrow-based MRD by next generation sequencing (NGS). Concordance between MALDI-TOF and BM NGS was high, although LC-MS consistently seemed to be more sensitive at detecting residual paraprotein, even among cases that were MRD-negative by other assessments. Somewhat surprisingly, when assessed at the end of cycle 8, none of the methods of MRD assessment in the bone marrow or peripheral blood met statistical significance for prognosis. A major reason for this might be due to sample size, given trends in favor of MRD-negativity were seen for each method, it may also be that cycle 8 is too early to assess for the prognostic significance of MRD when using extended duration of highly active myeloma therapy. Irrespective of statistical considerations, it seems quite meaningful that no patients who had either sustained MRD-negativity in the marrow or negative LC-MS at any time experienced disease progression.

Although the optimal time point to assess MRD remains to be determined, there is great promise for using peripheral blood MRD to assist with treatment decisions. It is noteworthy that in the current era of myeloma therapy, with potent and well-tolerated combinations, the optimal duration of induction might not be a uniform number of cycles. Perhaps there is an opportunity for MRD to assist with individualized decisions regarding duration of initial combination therapy, particularly if a peripheral blood assay could help to avoid serial invasive, painful, and labor-intensive BM biopsies. The potential applications of MRD in the management of myeloma are numerous, and research questions will continue to evolve in the coming years [8, 9].

In conclusion, Derman, et al. are congratulated for a well-conducted trial with extended DaraKRd. This regimen is feasible and highly effective at controlling myeloma for most patients. Whether this treatment strategy is preferred over alternative regimens will remain an open question, but we are fortunate to have gained insight into the kinetics of peripheral blood MRD assays and the natural history of myeloma with this treatment strategy.
